# Protocol for two-arm pragmatic cluster randomized hybrid implementation-effectiveness trial comparing two education strategies for improving the uptake of noninvasive ventilation in patients with severe COPD exacerbation

**DOI:** 10.1186/s43058-020-00028-2

**Published:** 2020-05-06

**Authors:** Mihaela S. Stefan, Penelope S. Pekow, Christopher M. Shea, Ashley M. Hughes, Nicholas S. Hill, Jay S. Steingrub, Peter K. Lindenauer

**Affiliations:** 1 Institute for Healthcare Delivery and Population Science, University of Massachusetts Medical School–Baystate, Springfield, MA USA; 2 Department of Medicine, University of Massachusetts Medical School–Baystate, Springfield, MA USA; 3 School of Public Health and Health Sciences, University of Massachusetts, Amherst, MA USA; 4 Department of Health Policy and Management, Gillings School of Global Public Health, University of North Carolina-Chapel Hill, Chapel Hill, NC USA; 5 College of Applied Health Science, University of Illinois at Chicago, Chicago, IL USA; 6 Division of Pulmonary and Critical Care Medicine, Tufts University School of Medicine, Boston, MA USA; 7 Division of Pulmonary and Critical Care, Department of Medicine, University of Massachusetts Medical School–Baystate, Springfield, MA USA; 8 Department of Population and Quantitative Health Sciences, University of Massachusetts Medical School, Worcester, MA USA

**Keywords:** COPD, Noninvasive ventilation, Interprofessional training, Education, Teamwork, Implementation strategies

## Abstract

**Background:**

COPD is the fourth leading cause of death in the US, and COPD exacerbations result in approximately 700,000 hospitalizations annually. Patients with acute respiratory failure due to severe COPD exacerbation are treated with invasive (IMV) or noninvasive mechanical ventilation (NIV). Although IMV reverses hypercapnia/hypoxia, it causes significant morbidity and mortality. There is strong evidence that patients treated with NIV have better outcomes, and NIV is recommended as first line therapy in these patients. Yet, several studies have demonstrated substantial variation in the use of NIV across hospitals, leading to preventable morbidity and mortality. Through a series of mixed-methods studies, we have found that successful implementation of NIV requires physicians, respiratory therapists (RTs), and nurses to communicate and collaborate effectively, suggesting that efforts to increase the use of NIV in COPD need to account for the complex and interdisciplinary nature of NIV delivery and the need for team coordination. Therefore, we propose to compare two educational strategies: online education (OLE) and interprofessional education (IPE) which targets complex team-based care in NIV delivery.

**Methods and design:**

Twenty hospitals with low baseline rates of NIV use will be randomized to either the OLE or IPE study arm. The primary outcome of the trial is change in the hospital rate of NIV use among patients with COPD requiring ventilatory support. In aim 1, we will compare the uptake change over time of NIV use among patients with COPD in hospitals enrolled in the two arms. In aim 2, we will explore mediators’ role (respiratory therapist autonomy and team functionality) on the relationship between the implementation strategies and implementation effectiveness. Finally, in aim 3, through interviews with providers, we will assess acceptability and feasibility of the educational training.

**Discussions:**

This study will be among the first to carefully test the impact of IPE in the inpatient setting. This work promises to change practice by offering approaches to facilitate greater uptake of NIV and may generalize to other interventions directed to seriously-ill patients.

**Trial registration:**

Name of registry: ClinicalTrials.gov

Trial registration number: NCT04206735

Date of Registration: December 20, 2019

Contributions to the literature

- This will be among the first studies to analyze the effectiveness and feasibility of implementing interprofessional education alongside a standard of care initiative (e.g., NIV use in COPD exacerbations) as a continuing educational course onsite at hospitals.
- This study will add to the literature regarding the role of interprofessional education on team functionality for a standard of care initiative that can impact the use of NIV, length of patient stay, and mortality for COPD patients.

## Background

Chronic obstructive pulmonary disease (COPD) is the fourth leading cause of death in the US, and COPD exacerbations result in approximately 700,000 hospitalizations annually [1, 2]. Patients who do not respond to pharmacotherapy are placed on invasive (IMV) or noninvasive mechanical ventilation (NIV). While invasive mechanical ventilation (IMV) administered through an endotracheal tube is an effective method of treating acute respiratory, it requires treatment in an intensive care unit, and places patients at risk for a wide range of complications, including ventilator-associated pneumonia. NIV (continuous positive airway pressure, CPAP or Bilevel positive airway pressure, BIPAP) provides positive pressure ventilation via a face mask without the need for intubation. Multiple randomized controlled trials [3, 4], meta-analysis [5, 6], and analyses of real-world data [7, 8] have demonstrated that treatment with NIV, when added to usual care, reduces the risk of intubation, lowers the incidence of ventilator associated complications, and results in better short-term survival. Based on this evidence, NIV receives a grade A recommendation in current **G**lobal Initiative for Chronic **O**bstructive **L**ung **D**isease (GOLD) guidelines [9]. Furthermore, the European Respiratory Society and American Thoracic Society joint guidelines [10] as well as British Thoracic Society guidelines [11] make a strong recommendation for the use of NIV as a first-line treatment for patients with COPD exacerbation and acute respiratory failure.

**Fig. 1:**
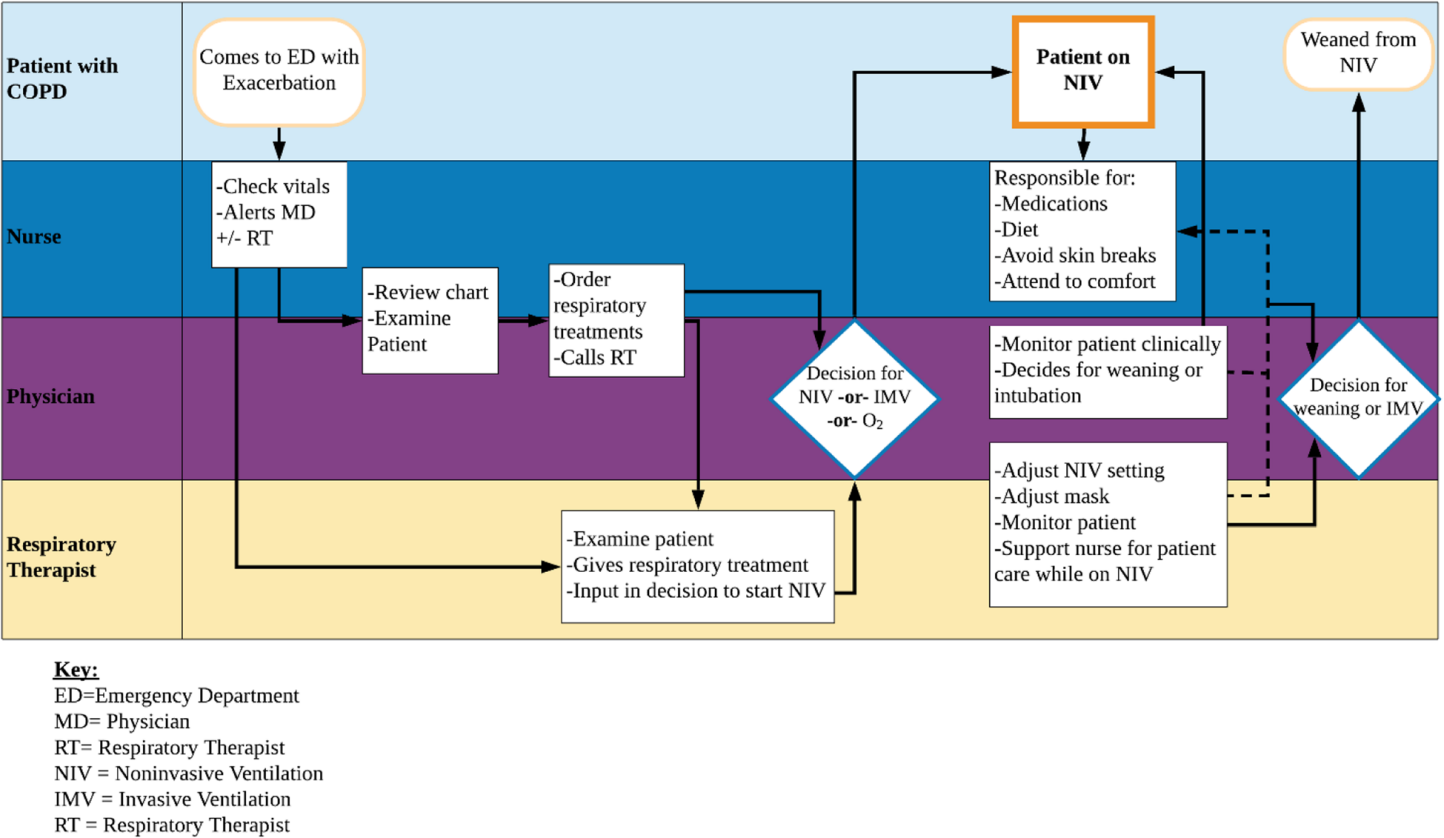
Clinician roles in management of patients with severe COPD in need for ventilation. Key: ED = Emergency Department, MD = Physician, RT = Respiratory Therapist, NIV = Noninvasive Ventilation, IMV = Invasive Ventilation, RT = Respiratory Therapist

Although the evidence supporting the use of NIV is compelling, prior research has demonstrated substantial variation in the use of NIV in routine clinical settings, highlighting a persistent gap in NIV adoption. In a recent study of more than 77,500 patients with COPD cared for at 400 US hospitals, median hospital percentage of NIV use among ventilated patients was 75.1% (range 9.2–94.1%) and the bottom 20% of hospitals offered a trial of NIV to less than half of ventilated patients [12]. More importantly, institutions with higher rates of NIV had lower IMV use and better clinical outcomes. Thus, low hospital rates of NIV in patients admitted for severe COPD represents an evidence practice gap and a missed opportunity to improve the outcomes among this vulnerable population. Appropriate delivery of NIV is a complex, multicomponent intervention that requires timely recognition, and effective communication, and coordination across multiple disciplines. Figure 1 depicts the flow for a patient who comes to the emergency department with severe COPD exacerbation and each clinician’s responsibilities in the process of NIV initiation and monitoring. Only few studies have tested strategies for supporting NIV implementation. A single site, before-after study from Canada found that multidisciplinary guidelines for the use of NIV in patients with acute respiratory failure (ARF) were associated with greater NIV utilization but included only patients in the intensive care unit [13].

**Fig. 2:**
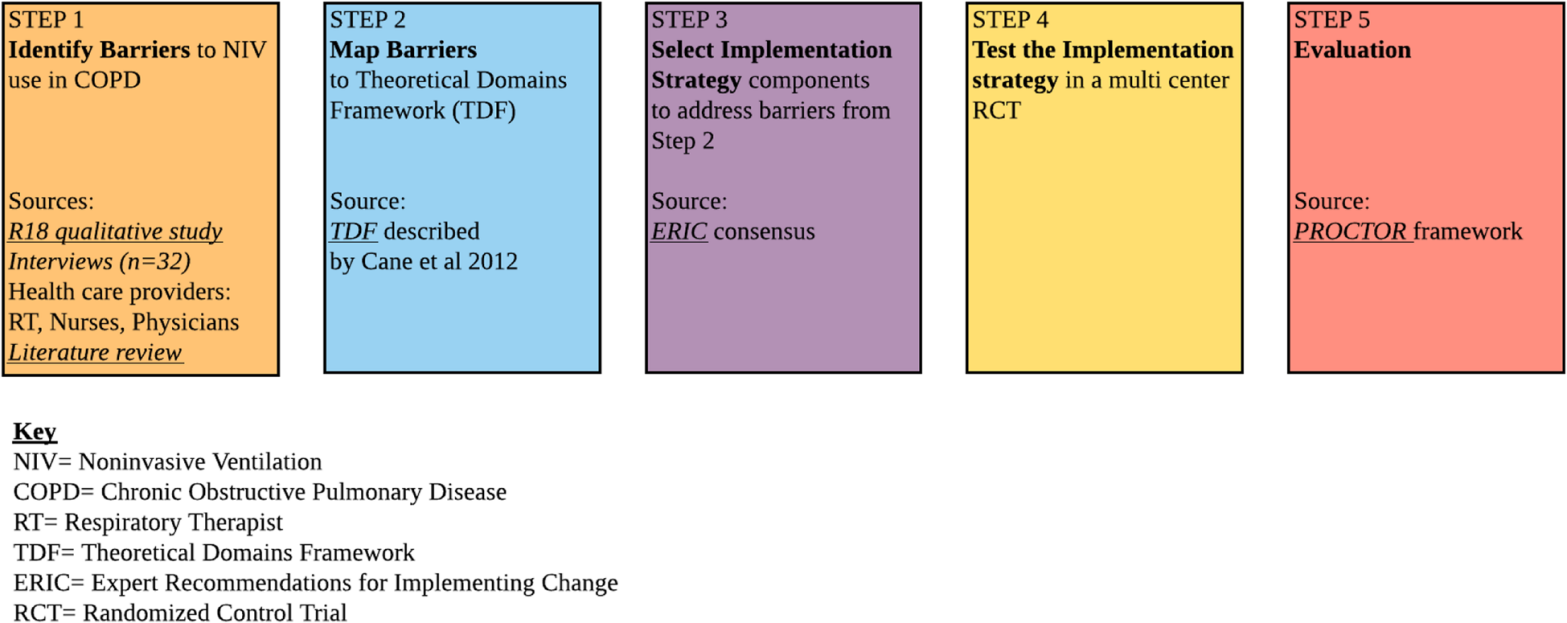
Step-by-step development and evaluation of the implementation strategy. Key: NIV = Noninvasive Ventilation, COPD = Chronic Obstructive Pulmonary Disease, RT = Respiratory Therapist, TDF = Theoretical Domains Framework, ERIC = Expert Recommendations for Implementing Change, RCT = Randomized Control Trial

For this study, we used the intervention mapping process model to develop and select implementation strategies to increase the uptake of NIV [14]. Figure 2 summarizes the steps in the development of our implementation strategy to increase the use of NIV in severe COPD exacerbation. In step 1 (formative evaluation), we conducted semi-structured interviews with key informants in a sample of hospitals with high rates of NIV and good COPD outcomes (low mortality and NIV failure rates). The analysis of the interviews revealed 3 different professional identities and roles in NIV delivery: physicians, respiratory therapists (RTs), and nurses. Although several clinicians’ tasks are distinct, Fig. 1 shows the connections between physicians, nurses, and RTs indicating the need for coordination to ensure optimal patient outcomes. The three groups encounter significant professional boundary issues with regards to their work responsibilities and priorities. For example, nurses were concerned about patient’s inability to eat or take medications while on NIV. RTs considered that nurses do not have a good understanding of the vital role of NIV. However, the two professions agreed that when there was a shared understanding of the treatment plan and when the concerns from both sides were openly addressed, the collaboration was considerably improved. RTs perceived themselves as experts in initiating and managing NIV; in some institutions, there was a strained relation between the RTs and physicians, with RTs complaining about a lack of autonomy and the need to wait for physicians when NIV was immediately indicated. We identified the following contextual factors and strategies associated with successful NIV implementation: provider buy-in, respiratory therapists (RT) autonomy to deliver NIV independently, interdisciplinary teamwork, collegial, trusting relationships between RTs, physicians, and nurses, and ongoing staff education [15].

In step 2, we organized the specific types of determinants that influence NIV delivery using the Theoretical Domain Framework (TDF) [16]. The TDF was used as a guiding theory for this project because the desired behavior change is primarily at the individual level, e.g., convincing providers to consider NIV in any patient with severe COPD exacerbation. We summarized the barriers in the TDF domains with an eye toward choosing an implementation strategy which could overcome several of the identified barriers. Eight of the 14 domains (knowledge, skills, professional roles and identity, beliefs about capabilities, beliefs about consequences, environmental context and resources, social influences, and emotion) were present in the existing literature and our research.

Interdisciplinary teamwork, on-going education, providers buy-in, and RTs autonomy were found as the top four determinants for successful NIV delivery in COPD exacerbations. These findings suggest that to succeed, implementation strategies need to account for the complex and interdisciplinary nature of NIV therapy and the need for team coordination.

Step 3: to guide the selection of implementation strategies, we used the Expert Recommendations for Implementing Change (ERIC) [17]. The main themes that emerged from the qualitative analysis and literature review mapped to the TDF domains and the implementation strategies most likely to address those barriers are shown in Table 1. Systematic analysis of the barriers suggested that to succeed, implementation strategies for knowledge transfer need to account for the complex and interdisciplinary nature of the NIV therapy and the need for team coordination; however, these hypotheses need to be carefully tested. Interprofessional, dynamic team training for physicians, RTs, and nurses was the implementation strategy selected as targeting several key determinants [16, 18, 19].

**Table 1:**
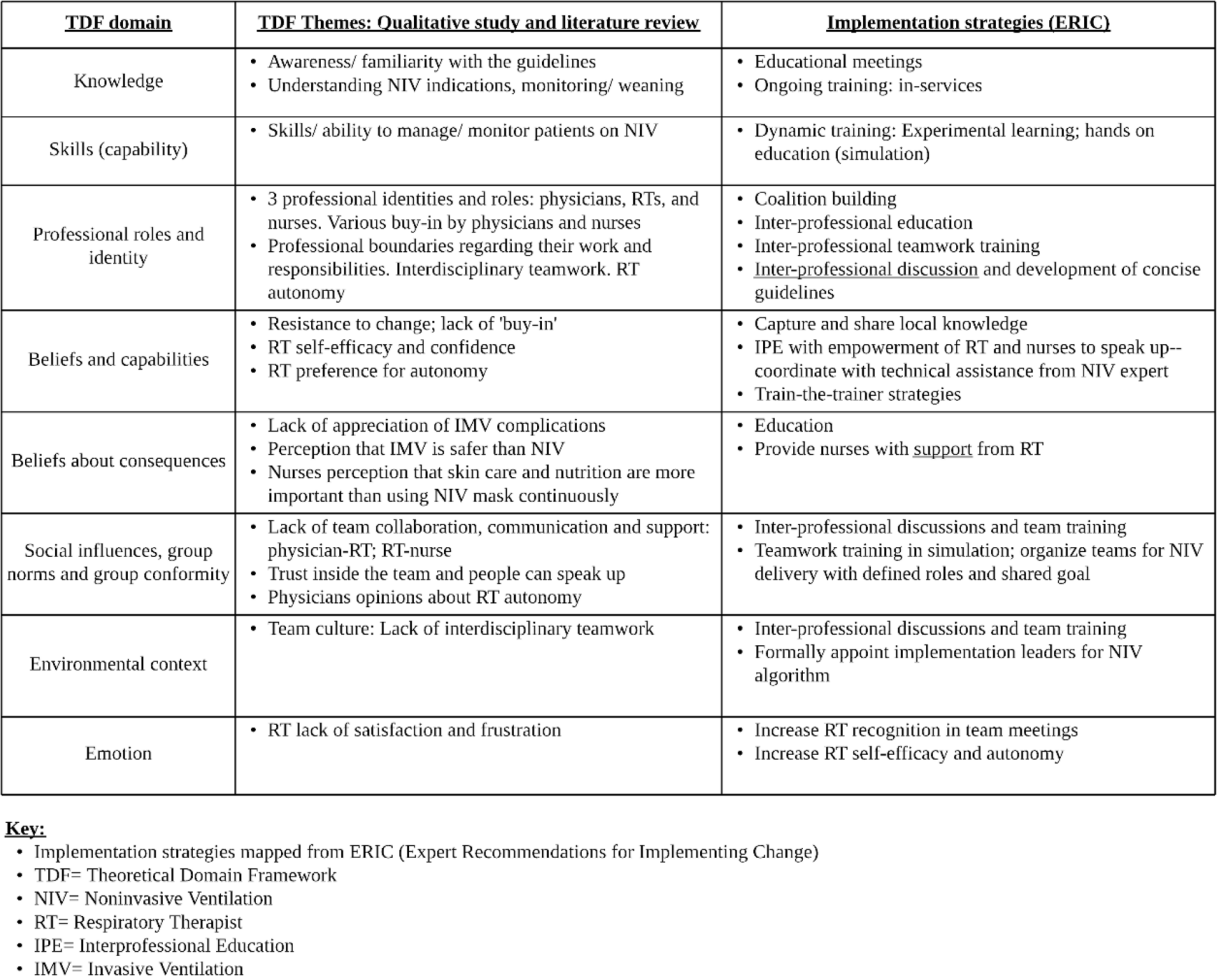
Themes mapped to theoretical domain framework and implementation strategies

NIV is delivered in high acuity environments by teams in which membership is dynamic, decisions must be made quickly, and members are not always face-to-face (asynchronously taking care of patients). This creates a critical need for effective communication, conflict management, and shared mental model [20] skills that are well suited to IPE approaches [21–23]. By contrast, conventional education regarding NIV is administered to individual clinicians or groups of clinicians of the same discipline via lectures or online modules. Educating individual care providers in silos does not address the interprofessional collaboration inherent to NIV delivery. On the other hand, IPE competencies emphasize the importance of establishing awareness and knowledge regarding interprofessional team roles. In this way, leveraging an IPE platform enables learning via interaction between two or more professions who learn from, with, and about each other’s roles and responsibilities (in this case, in regards to NIV) [24, 25].

The overall objective of this study is to conduct a pragmatic, parallel, 2-arm randomized cluster trial to compare the effectiveness of two implementation strategies: on-line education (OLE) and interprofessional education (IPE) on the uptake of NIV. The central hypothesis is that IPE will outperform conventional education, and that RT autonomy and/or team functionality will act as mediators. We will accomplish this goal by completing three specific aims.

Aim 1: To compare the effectiveness of OLE and IPE for increasing the delivery of NIV in appropriate patients hospitalized with COPD exacerbation.

Aim 2: To examine the effect of OLE and IPE on RT autonomy and team functionality as potential mediators of NIV uptake.

Aim 3: To evaluate the acceptability and feasibility of the OLE and IPE strategies to inform further refinement of the strategies.

## Methods and design

### Study design

In this cluster randomized controlled 2-arm parallel trial, 20 hospitals will be randomized to OPE or to IPE. Patients and clinicians are clustered within the hospitals because the IPE encourage facility-level change in clinicians’ communication and care coordination.

### Hospital recruitment

**Fig. 3:**
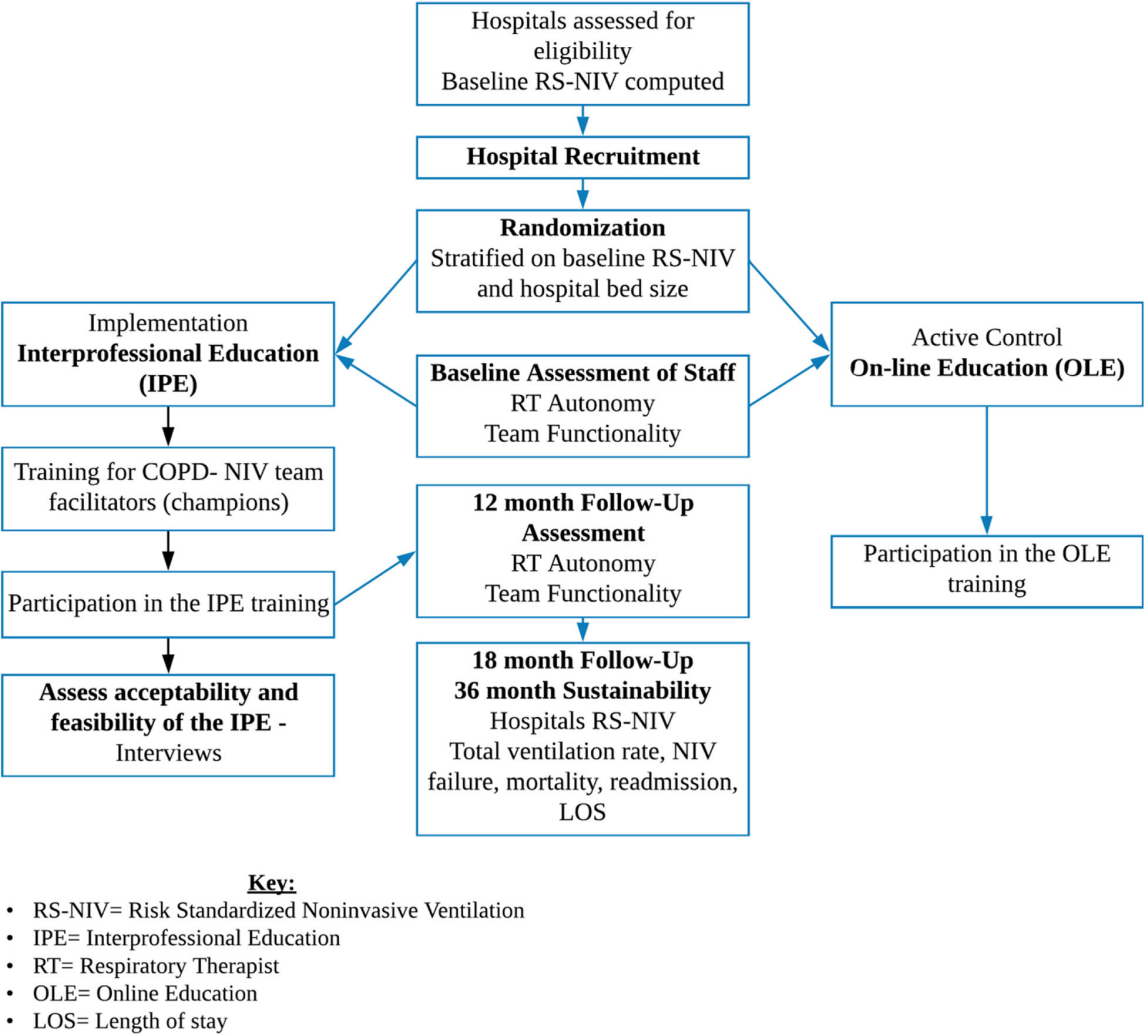
Study flowchart. Key: RS-NIV = Risk Standardized Noninvasive Ventilation, IPE = Interprofessional Education, RT = Respiratory Therapist, OLE = Online Education, LOS = Length of stay

The study will be conducted in 20 hospitals with risk-adjusted NIV proportion below median that have at least 35 eligible COPD admissions in an 18-month period. Potential eligible hospitals are those participating with data in Premier database, a voluntary, fee-supported database containing highly detailed hospital billing data pooled from more than 600 geographically and structurally diverse hospitals whose makeup closely resembles that of US hospitals. Hospitals that demonstrate interest in participating in the study will be asked to commit to form a COPD-NIV team composed of one physician, one RT, and one nurse that will be in close contact with the investigators and are responsible for delivering the educational intervention in their institution. Eligible hospitals will be contacted in a random order until the sample of 20 hospitals is achieved. To assess for potential participation bias, we will compare participating and refusing hospitals using available data such as size, ownership, teaching status, and location. The overall study design is shown in Fig. 3.

**Fig. 4:**
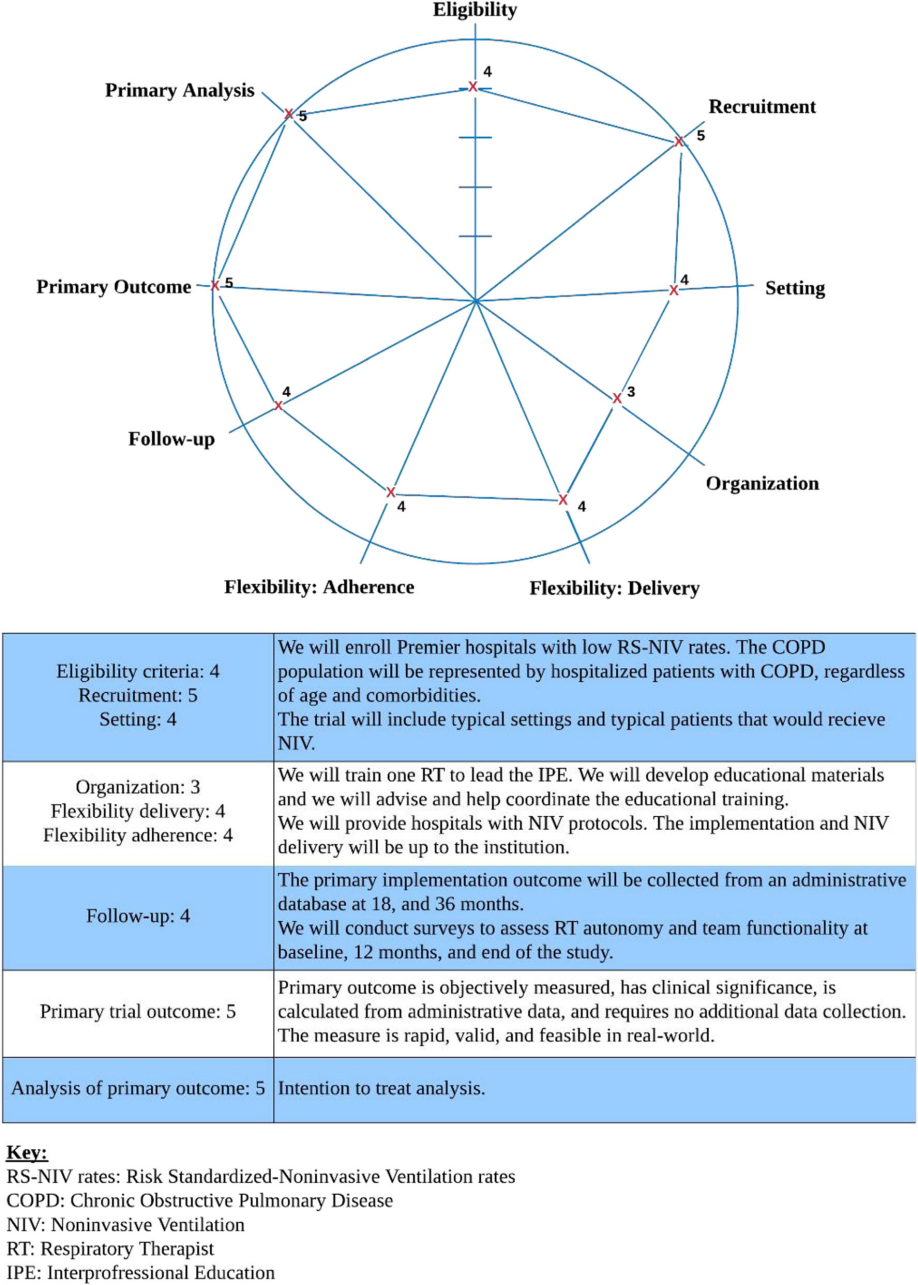
The explanatory continuum indicator summary (PRECIS). Key: RS-NIV rates: Risk Standardized-Noninvasive Ventilation rates, COPD: Chronic Obstructive Pulmonary Disease, NIV: Noninvasive Ventilation, RT: Respiratory Therapist, IPE: Interprofessional Education

The Explanatory Continuum Indicator Summary **(PRECIS**) framework was used to assess the pragmatism of the trial (Fig. 4).

### Randomization

We will randomly allocate hospitals to one of the study arms, stratified by the baseline NIV proportion and hospital bed size. A researcher not involved in the study and blinded to the identity of the hospitals will use a computer-generated randomization scheme. The randomization scheme will be concealed to the investigators. Due to the character of the intervention, it will not be possible to blind participants or investigators providing the educational program; however, the investigators will not be aware of the results of the study by intervention arm until the analysis is finalized.

### Implementation strategies

The trial will compare two implementation strategies: one active control consisting of traditional, online education (OLE) learning and a strategy of in-person interactive inter-professional education (IPE). Consent for participation in the educational strategies will be sought as a waiver of consent via an email sent to all potential participants after the randomization period and prior to the implementation at each site. Participants in the COPD-NIV teams and the training will be physicians, RTs, and nurses who are involved in treating patients with severe COPD exacerbation.

The following domains will be targeted in the educational training interventions:

1. Knowledge: evidence for NIV use, understanding patient selection, monitoring, and weaning (NIV algorithm); NIV advantage over IMV (OLE and IPE)
2. Skills: ability to manage/monitor patients on NIV: NIV settings, how to attend to patient comfort (OLE and IPE)
3. Interprofessional collaboration competencies: roles and responsibilities, teams and teamwork, values and ethics, and interprofessional communication (IPE only)

The hospital-based COPD-NIV teams will be responsible throughout the trial period for encouraging clinicians from each specialty to complete the courses. The investigators will have a conference call with the COPD-NIV team after the institutions have been randomized to discuss recruitment and surveys’ delivery. (e.g., RT Autonomy, team functionality, and organizational readiness for implementing change). Conference calls between the investigators and the individual COPD-NIV team will continue every quarter for the duration of the 18 months of active implementation period with a follow-up call at the end of the study.

#### Active control group: online education (OLE)

Sites will be given access to free continuing education modules customized for each discipline; RT and nurse online education training will be approximately 30 min long, and physician’s online education training will be about 1 h. The modules will be delivered online through a secure website that will allow us to count the number of providers completing the course. We will use traditional PowerPoint presentations with embedded whiteboard animation videos. We selected an active control instead of usual care, because it will allow stronger inferences about the benefits of IPE when compared to more traditional learning approaches. The online modules will be offered for the entire period of the study for all the new staff.

#### Intervention group: interprofessional education (IPE)

It will consist of a 60-minute in-person interprofessional educational workshop.

##### Training of the facilitators

We will organized a one day in-person training for all the NIV-COPD teams. The training will consist of 2 modules: (1) NIV knowledge and skills: delivered by NIV experts and (2) IPE: delivered by an expert in IPE and team training. The didactic training regarding NIV use in COPD will include a review of the evidence supporting the benefits of NIV, advantages of NIV as compared to invasive mechanical ventilation (IMV), selection of patients, contraindications to NIV, and management of patients while on NIV including monitoring, ventilator settings, attention to patient comfort, weaning from NIV, and decision about NIV failure and need for intubation. The didactic module will emphasize the importance of early initiation of NIV in patients with severe COPD. The second half of the training will concentrate on teaching interprofessional collaboration. Specifically, the Interprofessional Education Collaborative (IPEC) recommends four key competencies in successful IPE, which include roles and responsibilities, teams and teamwork, values and ethics, and interprofessional communication [26]. We selected the following core competencies for our interactive IPE. *Professional roles and identity:* each team member learns about abilities, tasks, duties, responsibilities, and concerns of their fellows’ team members; *values/ethics*: work with individuals of other professions to maintain a climate of mutual respect and shared values; *teams/teamwork*: apply relationship-building values and the principles of team dynamics to perform effectively in different team roles to deliver patient-center care that is safe, timely, effective, and efficient [27]. Given the demands for team coordination in NIV delivery and findings in our qualitative work, we anticipate that IPE will contribute to greater RT, physician, and nurse understanding of each other’s roles, increase team communication and functionality, and stimulate the development of shared mental models which facilitate coordination for NIV. We will use positional clarification which involves verbally presenting team members with information about their teammates’ jobs through discussion [28]. Psychological safety and speaking up will be encouraged and facilitated. For example, an RT may assume that physicians have more knowledge about NIV delivery for a particular patient than they do (because physicians are generally more knowledgeable about treatments) and remain quiet; when in fact, the RT has important information about how the patient may respond (cognitive bias) [29].

The sessions will be recorded, so that participants will have ongoing access to the content. We anticipate that the members of the COPD-NIV team who will become the training facilitators at their hospital will not be subject matter experts in the training context, especially the IPE. Therefore, a special instructor script will be written and will be paired with the presentation.

##### Delivery of the IPE sessions at institutions randomized to IPE

Training sessions for clinicians at IPE sites will be led by the COPD-NIV teams and will include information (e.g., 30-min lecture), demonstration (providers will be provided with contextualized examples), and practice (30-min, action-based approach with guided practice). It will contain a scenario of a patient with severe COPD coming to the emergency room with shortness of breath. They will review the guidelines for patient selection and monitoring and NIV settings and management. Each participant will be able to try the NIV mask and understand the importance of appropriate settings and attending to patient comfort. The three core competencies for interprofessional collaboration and how they apply to the NIV delivery will be explained. The IPE sessions will be offered up to twice a month for 3 months—the number will vary by institution depending on the number of providers that need to be trained. For the entire period of the study, we will continue to have every other monthly breakfast/lunch meetings where cases of patients with COPD in need of NIV will be presented with emphasis on interprofessional work structure, RT autonomy, and team functionality. Full IPE sessions will be offered once a month every 6 months for the new staff, as part of on-boarding.

### Outcome measures

Table 2 outlines the implementation and effectiveness outcomes at the cluster (hospital) level, their implementation timing, how they will be measured, and the source of data collection.

**Table 2:**
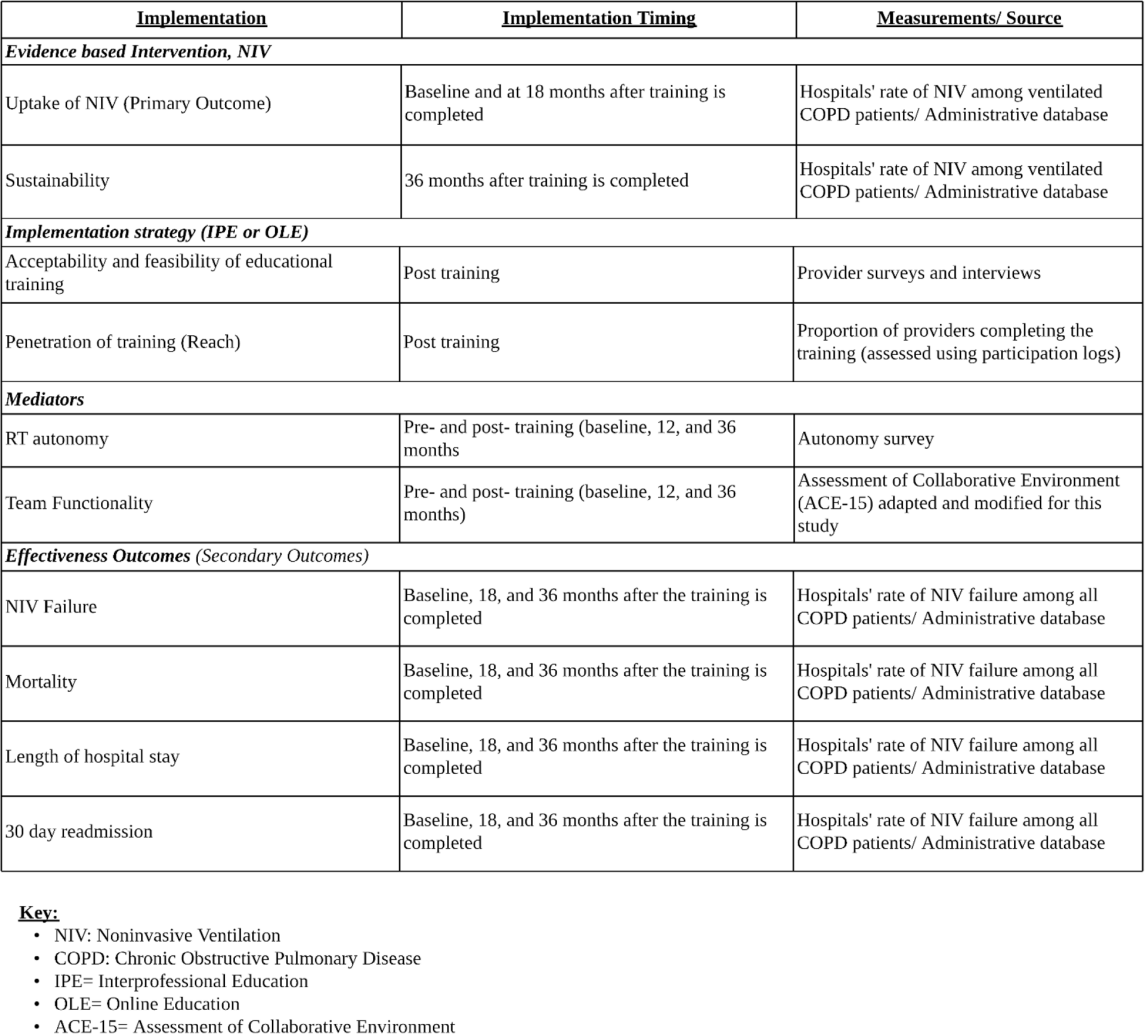
Outcome measures

### Analysis

#### Aim 1

To compare the effectiveness of OLE and IPE for increasing the delivery of NIV in appropriate patients hospitalized with COPD exacerbation.

##### Primary outcome

Hospital-level risk-standardized (RS) initial NIV proportion among patients hospitalized with a COPD exacerbation that were ventilated with NIV or IMV is assessed via administrative records of patients discharged from participating premier hospital who were 40 years or older and received a principal diagnosis of COPD, or a principal diagnosis of acute respiratory failure paired with a secondary diagnosis of COPD. We will use a previously validated set of ICD-10-CM codes that achieve a reasonable balance of sensitivity and specificity while minimizing potential biases [30].

##### Secondary outcomes

RS hospital rates of NIV failure (IMV after a trial of NIV), mortality, length of stay, and 30-day readmission among all patients with COPD.

All outcomes will be measured at the hospital level: (1) at baseline using prior 18 months of data, (2) at 18 months post-implementation to assess immediate/short term impact, and the following 18 months to assess sustainability. The 18-month assessment period is necessary to have adequate numbers of eligible COPD admissions for assessing hospital rates of NIV utilization. The time from randomization to the completion of the educational sessions with an expected duration of 3 months will not be included in the calculation of RS-NIV post-intervention. To examine penetration, we will measure providers’ exposure to educational training using participation logs.

##### Patient and hospital information

demographics, comorbidities, prior year number of admissions for COPD, prior year use of NIV or IMV, and outcomes will be identified from ICD-10 procedure codes and billing codes. For each participating hospital, we will record the number of beds, the annual number of admissions for COPD, teaching status, geographic region, and whether it serves an urban or rural population. We will also collect information about staffing: number of RTs, hospitalists, emergency room physicians, and nurses. We will record if hospitals use protocols for NIV initiation and management, and if NIV can be delivered on the regular medical floor or only in an intensive care unit. These factors will be used to describe participant hospitals and evaluated as potential confounders or effect modifiers.

##### Noninvasive and invasive ventilation

For each patient, we will examine standardized charge codes generated daily by respiratory therapists as well as dated ICD-10-procedure codes to determine whether or not they were treated with assisted ventilation, and, if so, whether ventilation was NIV or IMV. We define the primary method of ventilation as the first method by date and distinguish between patients treated with NIV as an initial strategy from those in whom NIV use follow exposure to invasive mechanical ventilation (IMV). We have previously validated the NIV ICD procedure codes and respiratory therapy charge codes by retrospective medical chart review. Using ICD-9-CM codes alone yielded a sensitivity of 86% (95% CI, 81–92%) and specificity of 92% (95% CI, 84–98%). The approach of using ICD-9-CM procedure codes and/or respiratory therapist charges increased sensitivity to 99% (95% CI, 98–4 100%) without reducing specificity (92%, 95% CI, 84–99%) [31].

##### Statistical analysis of aim 1

We will generate descriptive statistics overall, by hospital and educational strategy, including counts and percentages for categorical data, means, standard deviations, and percentile distributions for continuous data. We will compare characteristics of hospitals, including staffing characteristics, in the two study arms via chi-square tests and *t* tests or Wilcoxon tests. Characteristics of patients in the enrolled hospitals in the two arms will be compared via GEE models accounting for clustering by hospital. We will calculate the percentage of patients treated according to each of the primary ventilatory strategies: no assisted ventilation, NIV, and IMV. We will then calculate the proportion of patients initially treated with NIV among those who received assisted ventilation. We will estimate a risk-standardized proportion of ventilated patients initially treated with NIV (RS-NIV) for each hospital and for each data collection period (baseline period, 18 months post-intervention, and between 18 to 36 months post-intervention). We will use hierarchical logistic regression with a random hospital effect to model initial use of NIV among patients started on ventilation, adjusting for demographics, and comorbidities. From the model, a predicted NIV percentage for each hospital will be computed as the NIV percentage that would be anticipated at a particular hospital by using its hospital random effect, given the patient case mix. The expected NIV percentage will be computed as the rate that would be expected if the same patient mix were treated at an “average” hospital, using the average hospital effect. The RS-NIV percentage is then computed as the ratio of predicted to expected NIV percentage standardized by the overall unadjusted mean NIV percentage for all admissions in our model. Risk standardization has 2 key advantages: it adjusts for differences in patient mix, which may impact the suitability of NIV; it also provides more stabilized estimates based upon Bayesian shrinkage towards the overall mean among hospitals with small numbers of ventilated patients [32, 33]. The mean and median RS-NIV rates of the two arms will be computed for each study period. The primary analysis will use an analysis of variance model to compare IPE to OLE on change in RS-NIV rates from baseline to 18 months post-intervention. Additional analyses will adjust for hospital characteristics that are unbalanced between the study arms. A secondary analysis will compare the post-intervention levels, adjusting for baseline RS-NIV. To assess sustainability, similar models will be used to compare level of RS-NIV after an additional 18 months have passed.

For the secondary outcomes, our analysis will calculate hospital RS-rates of NIV failure, mortality, 30-day readmission, and length of stay among ventilated patients, as well as all COPD admissions for each study period. We will compare outcomes of OLE and IPE hospitals using models described above. Although we assume that the patients treated in IPE hospitals will have better clinical outcomes, we do not expect it to be able to detect an effect of these strategies on secondary outcomes due to overall low outcome rate, the small projected change, and relatively small number of clusters.

##### Organizational readiness

The implementation of the educational strategies to increase the use of NIV in COPD exacerbations will require the coordinated action of many organizational members (e.g., physicians, RTs, and nurses). The organizational readiness for implementing change (ORIC) survey can assess this construct at the supra-individual level (team, department, or organization) [34]. When organizational readiness is low, clinicians at these hospitals are likely to see the implementation (educational strategies) for the intervention (NIV use in COPD exacerbations) as undesirable and potentially avoid or resist planning for the implementation or participating in the implementation [34, 35]. The ORIC will allow us to identify the difference between organizations resisting the change (increasing NIV utilization) and sites that are unable to implement the educational strategies due to difficulties inherent in organizing and conducting the educational strategies at their hospital. To assess the readiness of the organization to implement the change, we will use a 7-item survey adapted from the original 10-item organizational readiness for implementing change (ORIC) [34]. This survey will measure change commitment and change efficacy of the organization towards increasing the rate of NIV use for COPD exacerbations.

##### Power and sample size for aim 1

The minimal number of hospitals participating in the trial was based on the analysis of Premier 2016–2017 data. Hospital RS-NIV proportions were calculated for the 457 hospitals with at least 35 eligible COPD admissions. The median RS-NIV rate among this group of hospitals was 82% (IQR 74–86%). We then selected the 48 hospitals with RS-NIV proportion less than 55%, based on the clinical impression that these hospitals would have sufficient room for improvement, as potentially eligible sites. Among these hospitals, the median number of eligible COPD patients was 197 over a 12-month period, ranging from 23 to 556 patients. The number of ventilated patients per hospital ranged from 7 to 163, with a median of 37. To achieve stability in estimation of hospital level RS-NIV, we will assess our primary endpoint at 18 months post-intervention expecting a minimum of 10 ventilated patients per hospital in which to assess NIV rates. Power analysis was conducted to determine the number of hospitals needed to assess the primary outcome of difference between the study arms in change in the hospital level risk-standardized proportion of ventilator starts that are NIV (RS-NIV). Using a type I error rate of 0.05, and standard deviation of change in rates over time derived from our prior work with the Premier data base, a total sample of 20 hospitals, 10 in each arm will give 80% power to detect difference of 15% in change (e.g., 5% increase among OLE hospitals, vs. 20% increase among IPE hospitals). Power is > 90% to detect a 20% difference in change between the intervention groups.

#### Aim 2

To examine the effect of OLE and IPE on RT autonomy and team functionality as potential mediators of NIV uptake.

##### Study design

To complete this aim, we will survey clinicians at baseline, 1 year, and end of the study period.

##### Participants and settings

We will select a random sample of 10 RTs for the RT survey and 21 providers (7 from each discipline) for team functionality and organizational readiness for change surveys. A waiver of consent will be sent to all potential participants via email, the survey link will be included at the end of the email. To maximize participation, we will provide $25 incentives to participants.

##### RT autonomy

Job autonomy is defined as the degree of perceived control that an employee has over how they perform tasks and the degree to which they operate independently. Prior studies showed that it mediates the relationship between employment status, work attitude, and performance [36, 37]. In our previous study, in-depth interviews with key stakeholders from a sample of hospitals with high use of NIV suggested that RT autonomy is critical to achieving timely initiation of NIV, often facilitated by the use of protocols [15]. These results are in line with prior literature [38] that supports the benefits of autonomous RT practice for weaning from IMV [39, 40]. Factors identified by the interviewees to contribute to RT autonomy were RT-driven protocols, RT expertise, and collegial relationship between RT and physicians. We assume that IPE will increase the physicians trust in RTs by allowing them to learn about their abilities and duties and concerns, and that IPE will facilitate team member recognition of their own knowledge with RTs being more likely to “speak up” (e.g., to suggest NIV use instead of intubation). To assess RT autonomy, we adapted a survey from the 11-item job autonomy measure from Aarons et al [37].

##### Team functionality

The IOM 2001 and 2006 quality chasm reports brought widespread attention to clinical teamwork as a means of improving safety and quality in healthcare [41]. Engagement in training-related activities designed to disseminate knowledge, skills, and attitudes for teamwork (such as IPE) is one way to acquire attitudes and behaviors consistent with teamwork and improve downstream impact on care quality and safety for patients [42–44]. get all clinicians (physicians, RTs, and nurses) involved in the delivery of NIV with the goal of promoting mutual trust and effective communication which will improve team functionality and hopefully promote NIV use. To assess team functionality, we adapted questions from the 4-point Likert scale Assessment of Collaborative Environment survey (ACE-15), a 15-item questionnaire which measures the perception of interprofessional “teamness” [45]. We created an 18-item questionnaire that has been divided into two parts: the first is the 9-item measure team functionality in managing patients with COPD exacerbation and the second is the 9–item measure team functionality in initiating NIV.

##### Statistical analysis

**Fig. 5:**
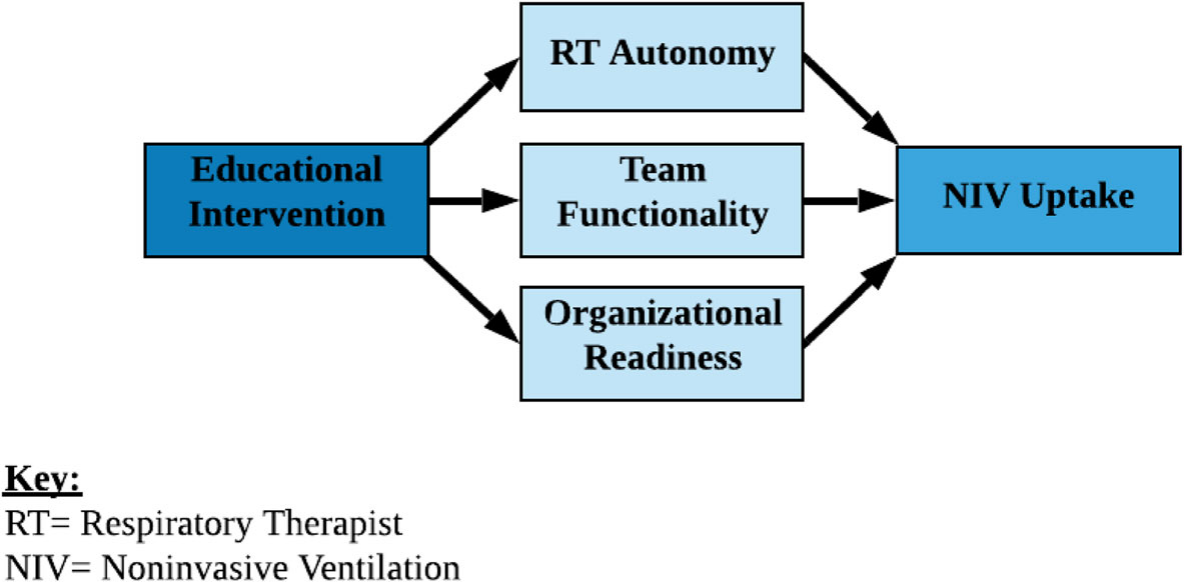
Mediation analysis. Key: RT = Respiratory Therapist, NIV = Noninvasive Ventilation

We will develop a series of models evaluating associations among the intervention, mediators, and outcome, including a structural equation model (SEM) to estimate the role of mediators as well as the direct effect of intervention on the outcome [46–49]. First, to evaluate the impact of educational intervention on the mediators RT autonomy and team functionality to implement change, multi-level models will be fit, clustering on hospital. Additional models will adjust for hospital and practitioner characteristics. Next, models for the primary RS-NIV outcome will be fit, with these potential mediators as the primary predictors. Main effects and interaction models will be evaluated. Then, the mediators will also be included as covariates in multi-level models including intervention, to evaluate whether RT autonomy and team functionality have effects on outcome, and after controlling for intervention. Finally, multi-level structural equation modeling will then be employed to estimate the indirect effect of the educational intervention on RS-NIV in the presence of mediators. Analysis will be performed using STATA’s gsem. This model will allow estimation of the direct effect of IPE intervention relative to OLE, in addition to the impact through the mediators (Fig. 5).

##### Power and sample size for aim 2

For aim 2 analyses, power was assessed for educational mode impact on job autonomy for respiratory RT measured by Aaron’s Job Autonomy Survey (AJAS), and team functionality measured by the ACE-15 tool. Estimating sample size to achieve 80% power, using a type I error rate of 0.05, a sample of 10 RT’s per hospital, will allow us to detect a moderate (Cohen’s *d* = .4) difference in AJAS at a 1-year post-intervention. This is accounting for clustering on hospital with intraclass correlation (ICC) in the range of .10–.20. Based upon data from local area hospitals, we estimate staffing of 8–10 RTs per 100 beds. For a few smaller hospitals, we will hope to include all RTs and may fall short of 10. Similarly, for the ACE-15, a sample size of 21 clinicians (7 each of RTs, RNs, and MDs) will achieve 80% power to detect a moderate effect size difference, accounting for clustering within hospitals, with ICC in the range of .10–.20 [50].

#### Specific aim 3

To evaluate the acceptability and feasibility of the IPE and OLE strategies and to inform further refinement of the strategies.

##### Study design

To achieve this aim, we will perform a qualitative study using semi-structured interviews with providers to assess relative importance of various barriers and determinants on the implementation of the two strategies.

##### Participants and settings

We will recruit a random sample of nurses, physicians, and RTs. As it is typical in qualitative research, the total sample is not fixed; depending of the size of the program, we will select 2–4 individuals from each profession in each hospital and expect to enroll approximately 40–50 providers enabling us to reach thematic saturation. Potential participants will be contacted via email by the study research assistant and invited to conduct an interview. A waiver of consent will be included in the body of the email prior to the contact information for the interview. Those who are interested in participating will be contacted by telephone to arrange an interview time over the telephone. To maximize participation, we will provide $50 incentives and schedule sessions at a time convenient to the participants.

##### Data collection

We anticipate that interviews will last approximately 30 min. All interviews will be audio recorded and transcribed verbatim. The interview team will consist of one research associate who will be trained by the investigator. The focus of the interviews will be to explore the implementation process—the acceptability and feasibility of the educational training strategies, participation in the training sessions, barriers to participation, adaptation made to the training sessions, and how the NIV protocol was incorporated into the clinical workflow.

##### Expected outcomes

This qualitative analysis will allow us to gain a broader perspective on the process of implementation from the perspective of the participants. We expect to understand and identify barriers and facilitators and their relative importance for this implementation strategy to be the most successful. The knowledge gained from this aim will be important for further application and refinement of IPE for other therapies/interventions directed to the critically ill patients.

##### Data analysis

Transcripts will be reviewed by the interviewer for accuracy. Qualitative data management and analysis software (NVivo) will be used to organize and code the data [51]. The nature of the interviewee role and the setting in which they work will be summarized and reported by location. We will use directed qualitative content methods to analyze interview content, beginning with a coding framework based on our prior work [52, 53]. Coding will occur concurrently with the interviews to ensure that the interview guide is eliciting data related to the domains of interest. Two team members will be primarily responsible for coding and will be supervised by one co-investigator proficient in qualitative analysis. The first 2 interviews will be read by each researcher with the goal of agreeing on the use of the domains and or constructs. This codebook will be used for all interviews going forward, with the team meeting periodically to discuss the emergence of any new codes or to clarify the relevance of domains and constructs to the text. Each transcript will be coded twice, once by each researcher. Discrepancies will be discussed until consensus is reached. Through regular investigators meetings, we will generate overarching themes.

### Economic evaluation

The investigation will collect the following information to calculate the costs to initiate the intervention: (1) cost of the training of the COPD-NIV team in the IPE arm of the study and inclusive of salary/fringe costs of the implementation specialists travel to the one day training session, (2) cost of webinars inclusive of speaker costs and video recording costs, and (3) cost of the continuing educational credits provided to the clinicians attending the IPE sessions at their hospitals and to the COPD-NIV team for their attendance of the 1-day session and for teaching the COPD-NIV-IPE course at their hospital.

### Dissemination

We propose the following strategies for dissemination: (1) after study completion, we will host webinars to share the results with the participant hospitals; (2) we will develop a one-page information sheet with the results and conclusion of the study and distribute it on the Premier Inc. website; (3) we will develop a toolkit and implementation manual with step-by-step guidance to help other institutions implement the IPE strategy; (4) publications and presentations at national and international respiratory and D&I conferences; and (5) we will work with Society of Hospital Medicine and COPD foundation to share the finding of the study to their members.

Table 3 present the proposed timeline for the study.

**Table 3:**
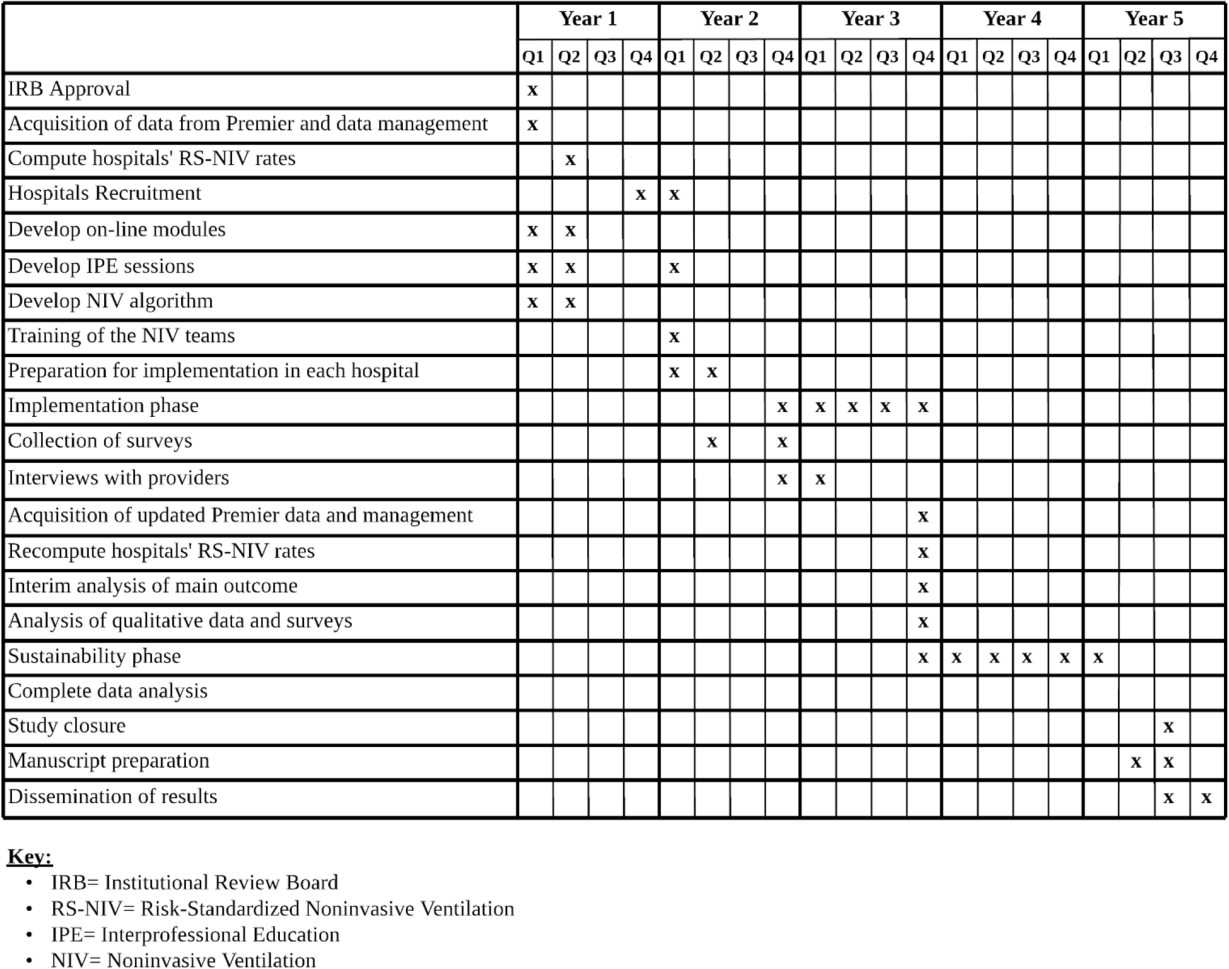
Proposed timeline

### Potential limitation and rational for key decisions

#### Why study only education?

We have carefully considered other implementation strategies such as audit and feedback, which is an electronic medical records decision support tool and academic detailing. When mapping the barriers within ERIC compilation of implementation strategies, we found that IPE covered several of identified barriers. Additionally, we were concern that using multiple strategies would complicate our attempt to understand the educational intervention impact. Interprofessional care is essential to the management of seriously ill patients and in the absence of robust studies to determine if IPE indeed impact patient outcomes, it is critical to be able to assess it in randomized controlled trial.

#### Why limit the trial to Premier hospitals?

For this pragmatic clinical trial, we need to be able to calculate hospitals’ NIV rates and identify low performing hospitals to be able to invite them to participate in the trial and determine the rates after the implementation period. Our intention was to have these rates calculated directly from the administrative or electronic data without a need for data collection. We did not come across any other database which provides the needs for this trial.

Still, if the trial shows that IPE is effective in improving the NIV rates, individual hospitals will be able to calculate their own rates and decide if they want to implement the IPE strategy.

## Discussion

This study will be among the first to carefully test the impact of IPE in the inpatient setting. Over the last 20 years, there have been increasing interest in linking IPE with interprofessional collaboration and team-based care [26, 54]; however, only recently have researchers begun to look beyond the classroom and beyond learning outcomes on such issues as patient safety, patient and provider satisfaction or quality, and cost of care. Consequently, the 2015 Institute of Medicine Report “Measuring the impact of interprofessional education on collaborative practice and patient outcomes” questions calls for purposeful, well-designed, robust studies to understand the link between IPE and patient and health systems outcomes [55]. Therefore, our study will add to the evidence by comparing an IPE strategy specifically designed to improve team functionality for NIV delivery in a pragmatic randomized controlled trial against an active, realistic control.

## Data Availability

Not applicable for this section

## Abbreviations

COPD: Chronic obstructive pulmonary disease
IMV: Invasive ventilation
NIV: Noninvasive ventilation
RT: Respiratory therapist
OLE: Online education
IPE: Interprofessional education
ARF: Acute respiratory failure
TDF: Theoretical domain framework
ERIC: Expert Recommendations for Implementing Change
COPD-NIV Team: Chronic Obstructive Pulmonary Disease-Noninvasive Ventilation Team
IPEC: Interprofessional Education Collaborative
RS: Risk standardized
ICD-10-CM codes: International Classification of Diseases, 10^th^ Revision, Clinical Modification codes
ICD: International Classification of Diseases
ICD-9-CM: International Classification of Diseases, 9^th^ Revision, Clinical Modification
GEE models: Generalized Estimating Equations model
ORIC: Organizational readiness for implementing change
IOM: Institute of Medicine
ACE-15: Assessment of Collaborative Environments
SEM: Structural equation model
AJAS: Aaron’s Job Autonomy Survey
ICC: Intraclass correlation
COPD-NIV-IPE: Chronic Obstructive Pulmonary Disease- Noninvasive Ventilation-Interprofessional Education
D&I Conferences: Dissemination and Implementation Conferences
